# Experimental evolution of competing bean beetle species reveals long‐term reversals of short‐term evolution, but no consistent character displacement

**DOI:** 10.1002/ece3.6164

**Published:** 2020-03-13

**Authors:** Stephen J. Hausch, Steven M. Vamosi, Jeremy W. Fox

**Affiliations:** ^1^ Department of Biological Sciences University of Calgary Calgary AB Canada

**Keywords:** bean weevils, *callosobruchus chinensis*, *callosobruchus maculatus*, character displacement, competition, experimental evolution, microcosms

## Abstract

Interspecific competition for shared resources should select for evolutionary divergence in resource use between competing species, termed character displacement. Many purported examples of character displacement exist, but few completely rule out alternative explanations. We reared genetically diverse populations of two species of bean beetles, *Callosobruchus maculatus* and *Callosobruchus chinensis*, in allopatry and sympatry on a mixture of adzuki beans and lentils, and assayed oviposition preference and other phenotypic traits after four, eight, and twelve generations of (co)evolution. *C. maculatus* specializes on adzuki beans; the generalist *C. chinensis* uses both beans. *C. chinensis* growing in allopatry emerged equally from both bean species. In sympatry, the two species competing strongly and coexisted via strong realized resource partitioning, with *C. chinensis* emerging almost exclusively from lentils and *C. maculatus* emerging almost exclusively from adzuki beans. However, oviposition preferences, larval survival traits, and larval development rates in both beetle species did not vary consistently between allopatric versus sympatric treatments. Rather, traits evolved in treatment‐independent fashion, with several traits exhibiting reversals in their evolutionary trajectories. For example, *C. chinensis* initially evolved a slower egg‐to‐adult development rate on adzuki beans in both allopatry and sympatry, then subsequently evolved back toward the faster ancestral development rate. Lack of character displacement is consistent with a previous similar experiment in bean beetles and may reflect lack of evolutionary trade‐offs in resource use. However, evolutionary reversals were unexpected and remain unexplained. Together with other empirical and theoretical work, our results illustrate the stringency of the conditions for character displacement.

## Graphical abstract

In a laboratory experiment, two competing bean beetle species partition food resources, but do not evolve character displacement in resource use traits. Rather, they adapt to features of their environment other than interspecific competition, in part via evolutionary reversals: Short‐term evolution of some phenotypic traits reverses in the long term.

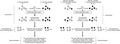

## 1 INTRODUCTION

Interspecific resource competition can select for competing species to evolve to use alternative resources for which there is currently less competition. The resulting divergence in resource use between competing species is known as character displacement (MacArthur & Levins, 1967; Stuart & Losos, 2013). Character displacement is thought to be an important mechanism generating and maintaining species diversity, particularly in species‐poor environments (Pfennig & Pfennig, 2010, 2012; Schluter, 2000).

In spite of its perceived importance, empirical evidence for character displacement is relatively scarce. Schluter and McPhail (1992) outlined six criteria that must be satisfied to infer that a putative case of character displacement actually is character displacement:

1. Chance must be ruled out as an explanation for the observed displacement.
2. Phenotypic differences between allopatric and sympatric populations must have a genetic basis.
3. Enhanced phenotypic differentiation between sympatric species must be due to evolution, not inability of phenotypically similar species to coexist.
4. Phenotypic differences should lead to differences in resource use.
5. Sympatric and allopatric environments should be the same.
6. Individuals with similar phenotypes should compete more strongly for shared resources than individuals with dissimilar phenotypes.

There are many purported examples of character displacement, but only eight cases, from just four taxonomic groups, meet all six criteria: threespine stickleback, Darwin's finches, Caribbean anoles, and *Escherichia coli* (Stuart & Losos, 2013).

The large gap between the numbers of purported and well‐established examples of character displacement likely is due in part to the difficulty in ruling out alternative explanations (Schluter & McPhail, 1992; Stuart & Losos, 2013). But another possibility is that character displacement may be a relatively uncommon outcome of interspecific competition. Theory predicts that competing species may coevolve convergent or parallel evolutionary changes in their resource use traits rather than divergence, depending on ecological and evolutionary details (Abrams, 1986, 1987a, 1987b; Fox & Vasseur, 2008). Theory also predicts that long‐term evolutionary changes in resource use traits may differ from short‐term changes even in the absence of abiotic environmental change (Fox & Vasseur, 2008). Short‐term character displacement thus might prove transient. Finally, organisms faced with resource competition (and resource scarcity due to other causes) ordinarily have many ecological and evolutionary options besides those assumed in character displacement theory. For instance, they can avoid resource competition by moving to another location, or evolve to require less of resources that are in short supply (Turner, Wade, Meyer, Sommerfeld, & Lenski, 2017).

Few previous studies used experimental evolution to study character displacement (Bailey & Kassen, 2012; Le Gac, Plucain, Hindré, Lenski, & Schneider, 2012; Taper, 1990; terHorst, 2011; Tyerman, Bertrand, Spencer, & Doebeli, 2008). Rather than asking whether putative cases of character displacement are actual cases, experimental evolution asks if character displacement evolves in conditions under which theory suggests it is most likely to evolve. Experimental evolution studies can be designed so as to satisfy the Schluter and McPhail (1992) criteria if character displacement evolves. And if character displacement does not evolve, the experiment can provide insight why not, thereby identifying limitations of existing theory and suggesting fruitful directions for future theory (Kawecki et al., 2012). Previous experimental evolution studies of character displacement obtained varying results (Bailey & Kassen, 2012; Le Gac et al., 2012; Taper, 1990; terHorst, 2011; Tyerman et al., 2008). Most relevant for our purposes is Taper (1990). Taper (1990) exposed the bean beetle *Callosobruchus chinensis* to interspecific competition from a mung bean specialist, *C. maculatus*, creating selection for *C. chinensis* to prefer a secondary resource (lentils) over its originally preferred resource (mung beans). Evolution resulting from resource competition was significant and equivalent to that produced by strong artificial selection (experimentally imposed zero survival on mung beans; Taper, 1990).

However, Taper (1990) has several important limitations. First, evolution in *C. maculatus* was not tracked, preventing parallel and divergent character displacement from being distinguished. Second, trait evolution was assessed only at the end of the experiment, preventing detection of temporal variation in the rate or direction of trait evolution. Third, Taper (1990) did not detect an evolutionary trade‐off in resource use; *C. chinensis* adapted physiologically to lentils, but without any cost to its physiological adaptation to mung beans. An evolutionary trade‐off in resource use is a key assumption in character displacement theory (Slatkin, 1980). Without an evolutionary trade‐off, all competing species would be expected to evolve into resource use generalists, without character displacement. The adaptation of *C. chinensis* to lentils in Taper (1990) thus might have evolved for other reasons besides interspecific resource competition (Tyerman et al., 2008). Fourth, Taper (1990) did not detect character displacement in the primary trait of interest, behavioral resource preference. *C. chinensis* did not evolve a preference for laying eggs on lentils rather than mung beans so as to avoid competition from mung bean specialist *C. maculatus*. Taper and Case (1992) hypothesized that failure to find character displacement in resource use preference in Taper (1990) might have been due to female avoidance of intraspecific competition. At high densities, female *C. chinensis* avoid laying eggs on beans with relatively more eggs already oviposited. The resulting uniform egg densities should reduce variation in fitness among females with different preferences for mung beans versus lentils, resulting in weak selection on resource use preference. Taper and Case (1992) also described a follow‐up study that was identified as “in preparation” at the time of writing but never published. Running their experiment for a further 16 further generations (under unspecified, modified conditions) confirmed the previous findings of physiological, but not behavioral, adaptation of *C. chinensis* to lentils, but also revealed an evolutionary trade‐off: Populations of *C. chinensis* growing in sympatry with the mung bean specialist *C. maculatus* developed slower on mung beans than did *C. chinensis* populations growing in allopatry.

Here, we describe a stronger test of character displacement in the *Callosobruchus* system. We reared genetically diverse populations of *C. chinensis* and *C. maculatus* in allopatry and sympatry on a mixture of adzuki beans (*Vigna angularis*; used by both species) and lentils (*Lens culinaris*; used by *C. chinensis*), assaying the traits of both species initially, and after four, eight, and 12 generations. We show that the system fulfills the ecological conditions expected to generate character displacement. We tested whether sympatric and allopatric populations diverged in their behavioral preference for lentils versus adzuki beans, and in their physiological adaptations to each resource. We predicted that *C. chinensis* growing in sympatry with *C. maculatus* would evolve increased behavioral preference for lentils and increased physiological adaptation to lentils, compared to *C. chinensis* growing in allopatry. We predicted the opposite changes for *C. maculatus*. We also compared the evolutionary trajectories of the two species when growing in sympatry to distinguish divergent, convergent, or parallel character displacement.

## 2 METHODS

Hausch, Fox, and Vamosi (2017) previously reported results of this experiment related to species coexistence. Here, we report results related to phenotypic trait evolution. Hausch et al. (2017) reported all methodological details except those related to trait assays, but we repeat those details here for ease of reference. The relevant text is edited from Hausch et al. (2017).

### 2.1 Study system background

We used the competing bean weevils *Callosobruchus maculatus* and *C. chinensis*. Adult female bean weevils mate and then oviposit on the surface of beans into which the larvae burrow and develop, eventually emerging as sexually mature adults. Adults do not feed (they do not need to and are not provided with food in laboratory conditions) and die 4–8 days after emergence. Our beetles had access to two food resources: adzuki beans (*Vigna angularis*), in which larvae of both species can consume and for which the specialist *C. maculatus* is the superior competitor under standard culture conditions (Hausch, 2015; Utida, 1953); lentils (*Lens culinaris*) are an alternative food resource used by *C. chinensis*, on which *C. maculatus* larvae experience high mortality and extremely slow development. In the context of this experiment, the two species thus exhibit a specialist‐generalist trade‐off (Taper, 1990), which in sympatry could select for the generalist *C. chinensis* to specialize on lentils and for the specialist *C. maculatus* to increasingly specialize on adzuki beans.

Competition for resources occurs in two distinct but related stages. First, adults compete to claim resources by laying eggs on beans. Second, the larvae compete with others in the same bean to consume the bean and develop faster. Comparing per‐bean densities of eggs versus emerging adults illustrates the intensity of competition among larvae within beans. Adzuki beans in established mixed cultures of *C. maculatus* and *C. chinensis* had an average of 48 eggs/bean oviposited on them, 89% of which hatched but from which only 6 adults/bean emerged on average (Hausch et al., 2017, S. Hausch, unpublished data). Lentils in established mixed cultures had an average of 14 eggs/bean, 79% of which hatched but from which an average of only 1.5 adults/bean emerged (Hausch et al., 2017, S. Hausch, unpublished data). We observed only scramble‐type larval competition, although contest competition (attacking other larvae) is well documented in *Callosobruchus* spp. (Toquenaga, 1993). Standard assays for contest competition, and x‐rays of beans, failed to identify any contest competition among our beetles (Hausch, 2015). The two species may also compete intra‐ and interspecifically via other mechanisms, such as egg mortality caused by high densities of adults damaging eggs as they move over bean surfaces, and mating interference of *C. chinensis* males with *C. maculatus* females (Fujii, 2009; Kishi & Tsubaki, 2014).

We obtained stock materials from Dr. Yukihiko Toquenaga at the University of Tsukuba, Japan. Each of our 10 lineages (five of each beetle species) originated from beetles collected from a different location, between 1940 and 2009 depending on the lineage, and has since been reared in the laboratory on adzuki beans. For 3 years before this experiment, we reared stock culture lineages separately from one another on adzuki beans in clear 22 × 12 × 8 cm containers with fine mesh lids allowing gas exchange. We maintained stock cultures on a discrete feeding schedule, providing approximately 40 g of adzuki beans every 23 days. This feeding schedule accommodated both early‐ and late‐hatching lineages. We removed old beans and dead adults approximately 14 days later. Stock cultures were initially synchronized through variations in rearing temperature. Stock cultures, treatments, and assays were all conducted in Percival I33LL environmental chambers at 30°C and 75% relative humidity in constant darkness. Environmental conditions were chosen to equalize the developmental rates of the two species. Adzuki beans and lentils were purchased commercially, frozen at −18°C for 48 hr, rinsed, and finally dried at 35°C. Beans that were noticeably wrinkled or split were not used. Adzuki beans were used if they passed through a 6 mm sieve but not a 3 mm sieve.

### 2.2 Pre‐experiment preparations

We created twenty populations of each of *C. maculatus* and *C. chinensis* by combining five conspecific lineages. These lineages vary phenotypically when grown in the same environment and thus differ genetically (Mano & Toquenaga, 2008; Takano, Toquenaga, & Fujii, 2001). This initial genetic diversity provided heritable standing variation on which selection could act. All ten lineages can exploit adzuki beans, but only some can exploit lentils. We raised lineages to equilibrium densities on 5 g adzuki beans. Combining involved aggregating 20% of the infested beans from each lineage during the larval stage. Initial lineage frequencies thus were proportional to their adzuki bean carrying capacities. To allow some genetic mixing, the high‐diversity populations were fed 5 g adzuki beans and 5 g lentils for one generation prior to initiating the experimental treatments. At the end of this generation, emerging adults were enumerated daily, recording their species identity and the resource from which they emerged.

### 2.3 Experimental design

The simplest experimental design would grow each species in allopatry and in sympatry with the other species. We chose a slightly more elaborate design to also test for effects of initial abundance in sympatry. Initial abundance might affect standing genetic variation and should mediate the strength of genetic drift, thereby affecting the potential for evolution by natural selection. We randomly assigned the 40 populations to four treatments with 10 replicates each: (a) *C. maculatus* alone, (b) *C. chinensis* alone, (c) *C. maculatus* (initially common resident) invaded by initially rare *C. chinensis*, and (d) *C. chinensis* (initially common resident) invaded by initially rare *C. maculatus*. Each species thus experienced three treatments: allopatric, initially common resident in sympatry, and initially rare invader in sympatry.

To start the experiment, we randomly assigned 10 populations of each species to grow allopatrically. Each of the other 10 populations of each species was divided into two subpopulations, comprising four (~15%) of the infested adzuki beans, and the remaining ~85% of the infested adzuki beans. Replicates of the M_res_C_inv_ treatment were produced by combining a small (four bean) subpopulation of *C. chinensis* with a randomly chosen large subpopulation of *C. maculatus*. Replicates of the C_res_M_inv_ treatment were produced by combining a small (four bean) subpopulation of *C. maculatus* with a randomly chosen large subpopulation of *C. chinensis*. Initially rare sympatric populations thus began at ~15% of carrying capacity, whereas initially common sympatric populations began at ~85% of carrying capacity.

**Figure 1:**
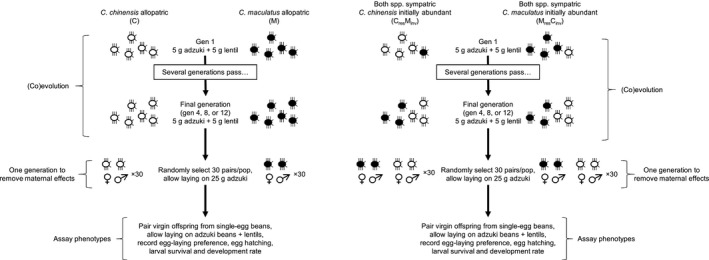
Schematic of the experimental design. White beetles represent *Callosobruchus chinensis*, filled beetles represent *C. maculatus*. Numbers of beetles are not indicative of actual densities in the experiment. Modified from Hausch et al. (2017)

The main experiment was maintained for 12 generations, each 23 d long, with species' phenotypic traits being assayed before the first generation (generation zero), and after the fourth, eighth, and twelfth generations (Figure 1). Assaying phenotypic traits was destructive: three, three, and four randomly chosen replicates from each treatment were removed for trait assays after the fourth, eighth, and twelfth generations, respectively.

For the duration of the experiment, communities were reared in 10‐cm diameter, quarter‐partitioned petri dishes under the same temperature, humidity, and light conditions as the stock cultures. Beetles were fed 5 g adzuki beans and 5 g lentils. On the first day of each generation, fresh beans were added into an unfilled quarter of the petri dish. On day 18 of each generation, dead adults were removed, identified to species, and enumerated. On the 18th day of generations four, eight, and 12, infested lentils and adzuki beans were separated by bean species. Emerging adults from each bean species were identified and enumerated daily, then either used in phenotypic trait assays (see following subsection), or else re‐aggregated into a single community to continue (co)evolving (Figure 1).

### 2.4 Phenotypic trait assays

We assayed the life‐history traits of a subsample of the populations after generations 4, 8, and 12. Traits were assayed in a common garden setting after one generation in isolation to reduce maternal effects (Figure 1). During this one generation, females were allowed to oviposit on adzuki beans without competition. Virgin male–female pairs were created from the F1 population, pairing individuals from the same population but different mothers. We assayed the oviposition behavior, survival, and development of the offspring of these pairings.

To create broodstock for the trait assays, we subsampled 30 unique male–female pairs per species from each population of each beetle species. Pairs were removed on the 3rd day of generation 4, 8, or 12 and provided approximately 25 adzuki beans (in a 60 mm petri dish) on which to oviposit for 24 hr. Pairs were assumed to not be virgin but the presence of males increased the likelihood that the female could lay a sufficient number of eggs. Pairs were returned to their population after the 24 hr. After seven days, once the eggs had hatched (with the larvae entering the bean) and turned opaque, eight infested beans were put into individual wells of a 48‐well plate. On average, 24 of the 30 pairs were successful broodstock (in others, one or both larvae died or eggs were inviable). Beans with only one egg were preferred but two‐egg beans were used if necessary (<1% of pairs). Secondarily, larger beans were preferred. Beans were checked every 24 hr for emerged adults and guaranteed virgins (females with no males in the same well or vice versa) were collected. Virgin females that had hatched out in the past 24 hr were paired with males from the same lineage that had hatched out in the past 24 (preferred) or 48 hr (<1% of pairs). Only one female was assayed from each broodstock pair, and the earliest emerging female with an available mate (on the day of emergence) was used. Occasionally, two males from the same broodstock pair had to be used to provide mates for the available females (12% of broodstock pairs). Mating pairs were placed in 60 mm petri dishes with 24 adzuki beans and 24 lentils. Mating pairs were allowed to mate and oviposit after which the adults were removed. After seven days, the beans were sorted by the number of eggs present.

Beans with only one egg were placed in individual plate wells to track development rate and survival, whereas beans with more than one egg were sorted by egg number and stored together to track average survival given larval density. One‐egg beans were checked every 24 hr, and the adult emergence date was recorded. Eggs with no emergence after 34 days were considered to have died. Beans with multiple eggs were incubated for 34 days, frozen, and then sampled for emerged adults. Restricting the maximum developmental time was necessary to prevent the fastest developers (18 days/generation) from hatching multiple generations.

### 2.5 Statistical analyses

Our analyses addressed three questions. First, does the system fulfill the ecological assumptions of character displacement generated by interspecific competition for shared resources (Schluter & McPhail, 1992)? Second, is character displacement observable at the end of the experimental evolution? Third, what was the evolutionary trajectory of the populations—continually increasing character displacement over time, or some more complicated trajectory?

Our experimental design automatically satisfies Schluter and McPhail (1992) criteria 3 and 5, respectively, no competitive exclusion and no difference between allopatric and sympatric sites. Checking whether our experiment fulfilled the other criteria for character displacement required data analysis. A negative relationship between the population densities of the two species was used to confirm criterion 6—species compete for resources. We regressed the density of *C. maculatus* against *C. chinensis* density, and vice versa, with a categorical variable for generation (nonsignificant interaction terms were removed). Both regressions excluded generation zero as densities in that generation were determined by our experimental design.

The ecological displacement due to competition (needed to test criterion 4—evolutionary displacement matches ecological displacement) was characterized by comparing the realized resource use and resource partitioning across treatments and generations. For each species, realized resource use was calculated as the proportion of adults emerging out of lentils (vs. adzuki beans; *N*_Lentils_/(*N*_Lentils_ + *N*_Adzuki_) × 100). Each species' resource use was compared across treatments in the 12th (final) generation using an ANOVA against the null hypothesis of no difference (prop. lentils = 50%). A priori contrasts were conducted between the two treatment conditions (resident and invader) and the allopatric control. For each community, realized resource partitioning was calculated as the average of the proportions of *C. chinensis* emerging from lentils and *C. maculatus* emerging from adzuki beans (i.e., average of *C. chinensis* realized resource use and 1 – *C. maculatus* realized resource use). Within the sympatric populations, a single‐sample *t* test was used to compare the grand‐mean realized resource partitioning to the null hypothesis of 0.5 and an ANOVA was used to test for differences in partitioning across treatments and generations.

For each species, we used an ANOVA to test for a treatment effect on assayed resource preference with a priori contrasts between the two treatment conditions (resident and invader) and the allopatric control. This analysis was initially restricted to the final generation but was repeated for *C. chinensis* with all treatment generations (generations 4, 8, and 12 as fixed, categorical effects) to confirm a marginally significant effect (*p* = .043). Including all treatment generations was deemed appropriate as the treatment‐by‐time interaction was nonsignificant (*p* = .6) and observations across generations are independent in our experiment.

Treatment differences in the final generation were tested for using MANOVAs with treatment, bean type, and their interaction (*C. chinensis*) or treatment alone (*C. maculatus*) as independent variables. A reduced model was used for *C. maculatus* because very few *C. maculatus* individuals oviposited on lentils in the assays, resulting in insufficient observations of *C. maculatus*' phenotype on lentils. We used the Pillai's Trace and, following Taper (1990), the Lawley–Hotelling Trace for our MANOVA test statistics. The dependent variables were larval survival, development rate, variation in development rate, egg survival, and number of eggs. For *C. maculatus*, uniformity of oviposition was also included. We followed the MANOVA results with ANOVAs for each dependent variables. Egg number was tested separately from the other traits because we were interested only in the interaction term; a significant bean‐by‐treatment interaction for egg number signifies a change in preference (Taper, 1990). For egg number, we tested the full model, with the interaction, and included all treatment generations for increased power. The remaining traits were tested with a series of orthogonal contrasts. First, the phenotypes on adzuki and lentil were compared with a bean contrast. Then, the invader and resident treatments were each compared to the allopatric control when considering only adzuki beans or lentils (four treatment contrasts). For *C. maculatus*, only the treatment contrasts on adzuki beans were used.

To understand the trajectory of evolution in our system, we tested for a change in the average phenotype between assays after accounting for treatment differences. For each trait‐by‐resource combination, we tested for evolution using an ANOVA of phenotype predicted by population, generation, and their interaction followed by a Tukey's post hoc test of the generation levels.

## 3 RESULTS

### 3.1 Ecological consequences of competition

**Figure 2:**
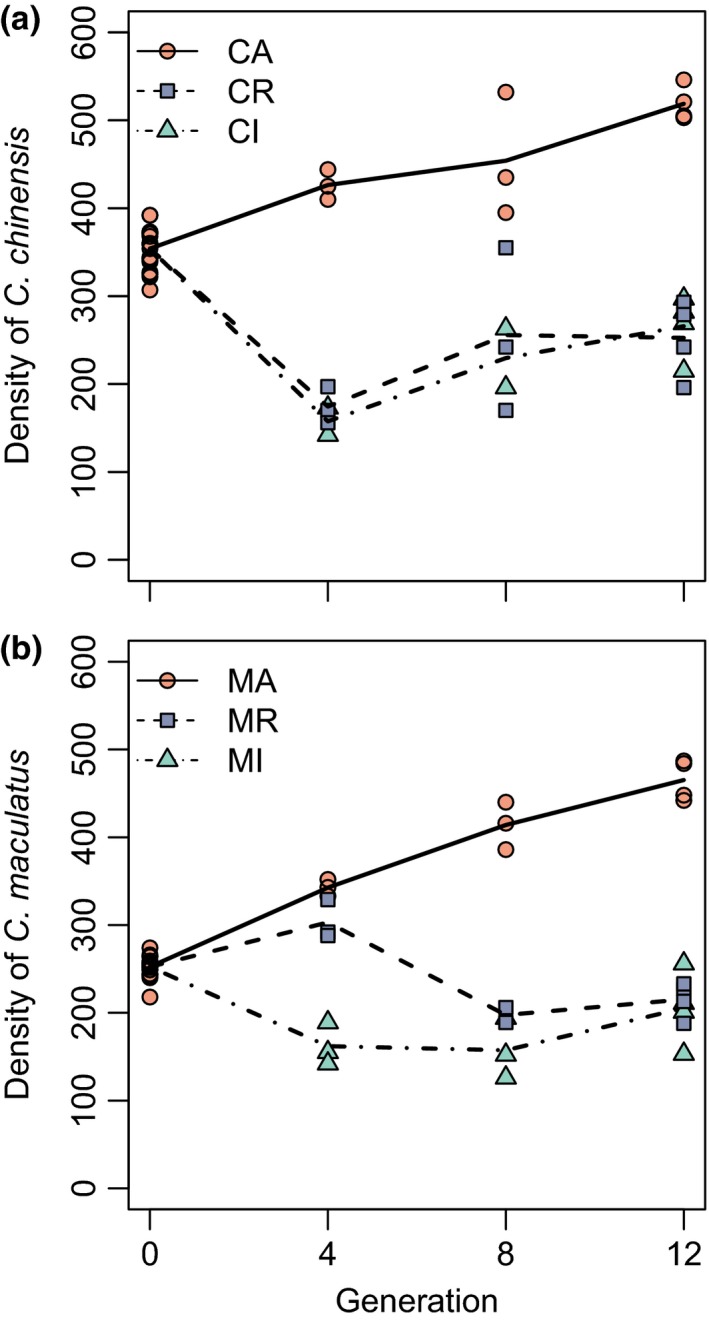
Abundances of *Callosobruchus chinensis* (a) and *Callosobruchus maculatus* (b) in the three treatments over time. Lines show variation over time in treatment means, and points are replicate observations. Orange circles and solid lines denote abundances in allopatry, blue squares and dashed lines denote abundances when growing as the initially abundant resident in sympatry, and green triangles and dash‐dotted lines denote abundances when growing as the initially rare invader in sympatry. CA, CR, and CI signify *C. chinensis* under allopatric, resident, and invader conditions, respectively. MA, MR, and MI signify the same but for *C. maculatus*

*Callosobruchus maculatus* and *Callosobruchus chinensis* competed; each species achieved lower density when growing in sympatry than when growing in allopatry (Figure 2). There was a linear and negative relationship between the two species and an increase in the system's total carrying capacity over time (regressions, *C. chinensis*: *F*_3,24_ = 54, *R* ^2^ = .87, *p* < .001; *C. maculatus*: *F*_3,25_ = 61, *R* ^2^ = .88, *p* < .001). Over the eight treatment generations, carrying capacity increased by approximately 25% (Figure 2; *p* < .001 for both species).

**Figure 3:**
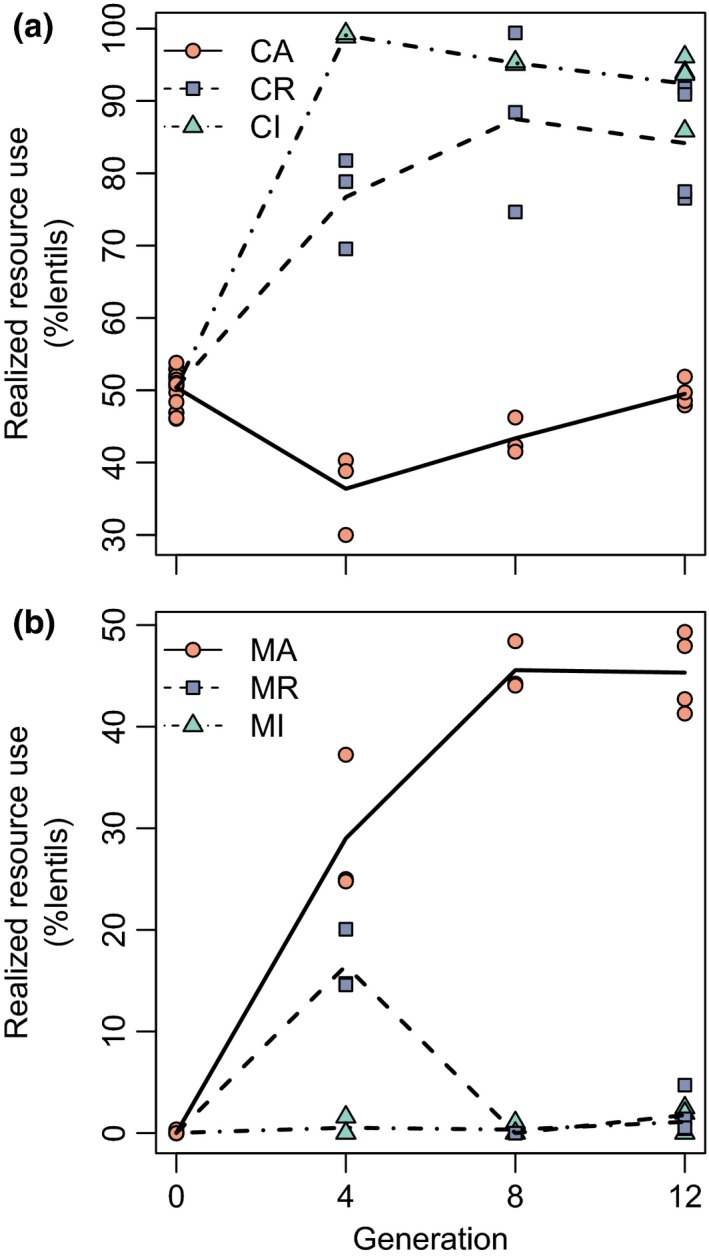
Change in realized resource use in the three treatments over time for *Callosobruchus chinensis* (a) and *Callosobruchus maculatus* (b). Lines show variation over time in treatment means, and points are replicate observations. Orange circles and solid lines denote resource use in allopatry, blue squares and dashed lines denote resource use when growing as the initially abundant resident in sympatry, and green triangles and dash‐dotted lines denote resource use when growing as the initially rare invader in sympatry. CA, CR, and CI signify *C. chinensis* under allopatric, resident, and invader conditions, respectively. MA, MR, and MI signify the same but for *C. maculatus*

Interspecific competition in sympatry led to realized resource partitioning, with each beetle species emerging primarily from the bean species on which it was expected to be the competitive dominant. Realized resource use, the percentage of individuals hatching out of lentils, varied significantly among treatments (Figure 3; ANOVA, *C. chinensis*: *F*_2,9_ = 68, *p* < .001; *C. maculatus*: *F*_2,9_ = 336, *p* < .001). By the final generation, *C. chinensis* growing in allopatry emerged equally from the two bean species (*β* = −0, *t* = −0.2, *p* = .863), and *C. maculatus* growing in allopatry showed only a small bias toward emerging from adzuki beans despite initially emerging almost exclusively from adzuki beans (*β* = −5, *t* = −3.5, *p* = .006). In sympatry, *C. chinensis* individuals emerged primarily but not exclusively from lentils, especially in the invader treatment where their initial density was low (resident vs. allopatric: *β* = 35, *t* = 9, *p* < .001; invader vs. allopatric: *β* = 43, *t* = 11, *p* < .001; Figure 3). In sympatry, *C. maculatus* continued to emerge from lentils in the fourth generation when resident, but was almost completely excluded from lentils by the final generation in both sympatric treatments (resident vs. allopatric: *β* = −44, *t* = −23, *p* < .001; invader vs. allopatric: *β* = −44, *t* = −24, *p* < .001; Figure 3).

### 3.2 (Co)evolution of oviposition preference

**Figure 4:**
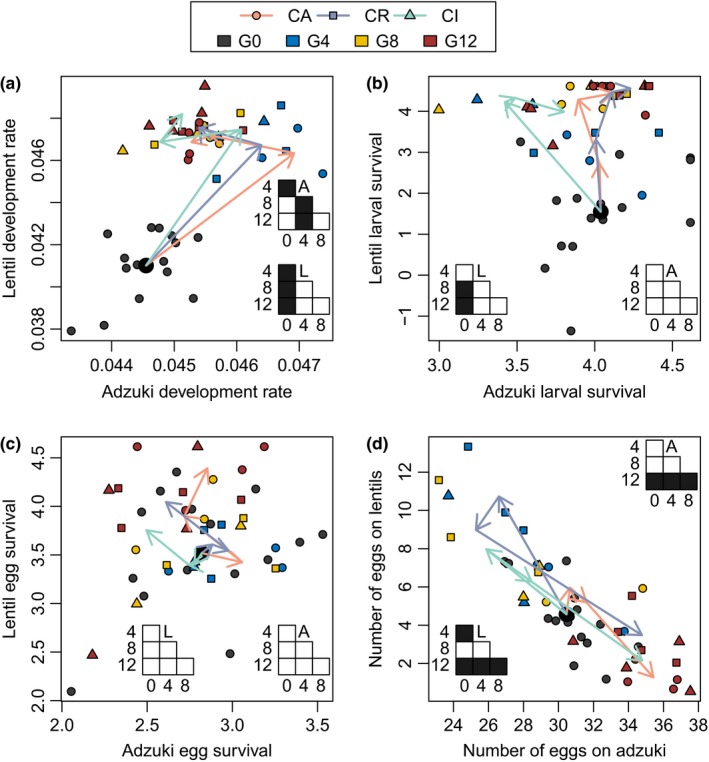
Change in *Callosobruchus chinensis* phenotypes in the three treatments over time. Each point denotes data from one replicate. Development rate traits have units of d^−1^ (i.e., inverse of development time in d). Larval survival traits are logit‐transformed larval survival probabilities. Arrows show changes over time in treatment means from generation 0 (G0; means denoted by large black circle), to 4 (G4, blue symbols), to 8 (G8, yellow symbols), to 12 (G12, red symbols). Orange arrows and circles denote *C. chinensis* trait evolution in allopatry (CA), blue arrows and squares denote *C. chinensis* trait evolution as resident (CR), and green arrows and triangles denote *C. chinensis* trait evolution as invader (CI). Grids summarize Tukey post hoc tests of the generation effect on the lentil (L) and adzuki (A) phenotypes. Filled cells are contrast pairs with *p* < .05, unfilled cells are *p* > .05

Generalist *C. chinensis* that coevolved with the adzuki bean specialist *C. maculatus* showed a greater common garden preference for laying eggs on lentils relative to the allopatric populations after 12 generations, although the difference was significant only when *C. chinensis* was the initially abundant resident in sympatry, not the initially rare invader (ANOVA, *F*_2,9_ = 2.8, *p* = .112; resident vs. allopatric, *t* = 2.4, *p* = .043; invader vs. allopatric, *t* = 1.4, *p* = .18; Figure 4d). The adzuki specialist *C. maculatus* did not exhibit a statistically significant difference in assayed egg laying preference between treatments after 12 generations (ANOVA, *F*_2,9_ = 1, *p* = .405; *p* > .25 for both contrasts).

*Callosobruchus chinensis* oviposition preference exhibited an evolutionary reversal over the course of the experiment. Averaging over the sympatric and allopatric treatments, *C. chinensis* oviposition on lentils increased significantly from the start of the experiment to the fourth generation, but subsequently decreased, so that by the final generation *C. chinensis* was ovipositing significantly fewer eggs on lentils and significantly more on adzuki beans relative to the initial levels (Figure 4d).

### 3.3 Adaptation to different resources

**Figure 5:**
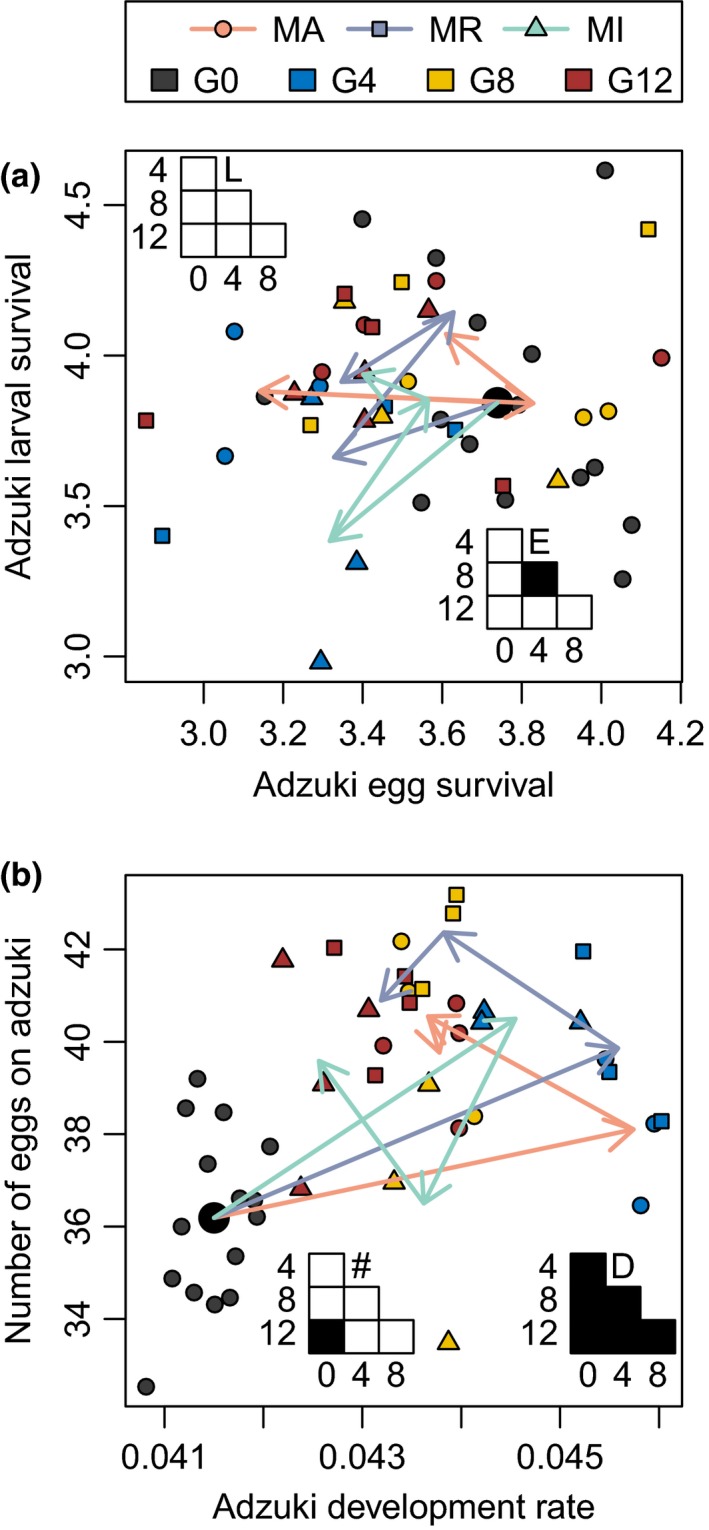
Change in *Callosobruchus maculatus* phenotypes in the three treatments over time. Each point denotes data from one replicate. Development rate traits have units of d^−1^ (i.e., inverse of development time in d). Larval survival traits are logit‐transformed larval survival probabilities. Arrows show changes over time in treatment means from generation 0 (G0; means denoted by large black circle), to 4 (G4, blue symbols), to 8 (G8, yellow symbols), to 12 (G12, red symbols). Orange arrows and circles denote *C. chinensis* trait evolution in allopatry (MA), blue arrows and squares denote *C. maculatus* trait evolution as resident (MR), and green arrows and triangles denote *C. maculatus* trait evolution as invader (MI). Grids summarize Tukey post hoc tests of the generation effect on the larval (L) and egg (E) survival, development rate (D), and number of eggs (#) on adzuki beans. Filled cells are contrast pairs with *p* < .05, unfilled cells are *p* > .05

*Callosobruchus chinensis* showed significant trait divergence between beans and treatments in the final generation (treatments: MANOVA, Pillai trace = 0.97, *df* = 10,30, *p* < .05; beans: MANOVA, Pillai trace = 0.978, *df* = 5,14, *p* < .001; Figure 4a–c). By the final generation, *C. chinensis* was better adapted to lentils than adzuki beans: Egg survival, within‐bean larval survival, and development rate all were significantly or marginally significantly higher on lentils than adzuki beans, while variation in development rate was significantly lower on lentils than on adzuki beans (ANOVAs; all *p* < .001 except within‐bean larval survival *p* = .057). Within‐bean survival rate of *C. chinensis* on both resources was significantly lower in invading sympatric populations relative to the allopatric populations (ANOVA, *F*_2,18_ = 4.9, *p* = .020; invader vs. allopatric, *t* = −2.4, *p* = .026). There was no statistically significant bean × treatment interaction on *C. chinensis* survival and development traits in the final generation (MANOVA, Pillai trace = 0.76, *df* = 10,30, *p* > .05). *C. maculatus* did not exhibit any statistically significant differences in survival or development traits among treatments in the final generation (MANOVA, Pillai trace = 1.17, *df* = 12,10, *p* > .05; Figure 5).

Both beetle species showed significant evolutionary change over the course of the experiment that was independent of treatment. For some traits, evolution in the first four generations partially or completely reversed in the subsequent eight generations (Figures 4 and 5). *C. chinensis* increased in development rate after four generations, emerging an average of 1 day faster on adzuki beans and 3 days faster on lentils relative to the initial population. *C. chinensis* subsequently sustained more rapid development on lentils, but its development rate on adzuki beans subsequently returned to near the initial rate (Figure 4a). *C. chinensis* larval survival on lentils, but not adzuki beans, increased significantly over the first eight generations (Figure 4b). *C. chinensis* egg survival showed no significant change over the experiment (Figure 4c).

Similar to *C. chinensis, C. maculatus* adults emerged from adzuki beans two days sooner on average after four generations but by the final generation were emerging only one day sooner (Figure 5a). *C. maculatus* oviposited significantly more eggs on adzuki beans by the final generation (Figure 5b). Unlike with *C. chinensis*, this increase represented a change in fecundity rather than preference. *C. maculatus* showed no significant change in larval survival on adzuki beans (Figure 5a) and only a transient change in adzuki egg survival (between generations 4 and 8; Figure 5b).

## 4 DISCUSSION

*Callosobruchus maculatus* and *Callosobruchus chinensis* compete strongly in our system and that interspecific competition leads to nearly complete realized resource partitioning. One might expect that this substantial realized resource partitioning would lead to strong selection in sympatry for each species to oviposit only on the beans on which its larvae are competitively dominant, since most eggs laid on other beans are doomed. And indeed, *C. chinensis* did initially evolve an increased oviposition preference for lentils—but this evolutionary change reversed in subsequent generations. By the end of the experiment, the expectation of character displacement was only partially satisfied at best: sympatric resident generalist *C. chinensis*, but not sympatric invaders, evolved a slight preference for ovipositing on lentils, relative to the allopatric populations. (Note that this result does not imply that sympatric resident and sympatric invader *C. chinensis* evolved significantly different oviposition preferences.) And the specialist *C. maculatus* evolved to use lentils much more often in allopatry than in sympatry, but did not evolve a corresponding divergence in oviposition preference between allopatry and sympatry. Lack of clear‐cut, long‐term character displacement in oviposition preference for either species confirms the results of Taper (1990) for *C. chinensis* and extends them to *C. maculatus*.

We can rule out lack of genetic variation and genetic drift as reasons for the lack of character displacement in oviposition preference, because oviposition preference (and other traits) exhibited substantial repeatable evolution over the course of the experiment. Lack of character displacement instead might reflect a weak or absent evolutionary resource use trade‐off. One might expect that substantial realized resource partitioning in sympatry would select for improved survival and development of each species on the bean species on which it is the competitive dominant, even at a cost to its ability to develop and survive on the other bean species. This expectation was not borne out. A resource use trade‐off in the generalist *C. chinensis*, in which adaptation to lentils comes at the cost of diminished performance on adzuki beans, was suggested for some traits, but depending on the trait was either not statistically significant or was not found in both sympatric treatments. It is also possible that assayed resource preference may not accurately predict oviposition behavior within a community (Taper, 1990).

Rather than evolutionary trade‐offs in resource use that would have manifested as different evolutionary trajectories in different treatments, we found substantial evolutionary change that was independent of treatment. Some of these treatment‐independent evolutionary changes likely represent adaptation to features of the culture regime that were common to all treatments. In particular, evolution of more rapid development rates likely represents adaptation to the 23 days generation length imposed by our experimental design. Possibly, an experiment that began with organisms already maximally well‐adapted to the culture regime might have revealed evolutionary trade‐offs that our experiment did not. We cannot entirely rule out the possibility that some treatment‐independent evolutionary changes actually represent batch effects of different batches of beans rather than evolutionary changes, but regard this possibility as unlikely.

We did find some modest differences in evolutionary trajectories between the sympatric resident and sympatric invader treatments. Some of these likely are attributable to the fact that, during the first few generations, sympatric residents experience primarily intraspecific competition, as in the allopatric treatments. However, others are more difficult to explain. In particular, it is unclear why sympatric resident *C. chinensis*, but not sympatric invader *C. chinensis*, would evolve slightly different oviposition preferences from allopatric *C. chinensis* by the end of the experiment. We cannot rule out founder effects in the sympatric invader populations. But their initial abundances were sufficiently high, and their evolutionary trajectories were sufficiently similar to those of the sympatric resident populations, that founder effects seem unlikely.

Interestingly, we found nonmonotonic evolution in both development rates and oviposition preference: Populations rapidly evolved—in the direction of character displacement, in the case of *C. chinensis* oviposition preference—but then reversed, evolving part or even all of the way back to the initial phenotype. Nonmonotonic evolution is unexpected on both empirical and theoretical grounds. Empirically, we know of few other examples of nonmonotonic evolution in the experimental evolution literature. Herron and Doebeli (2013) found nonmonotonic evolution was in the diversification of *E. coli* due to resource‐mediated coevolution. Theoretically, populations under selection to evolve toward a frequency‐independent optimal phenotype should approach the optimum monotonically, or with stochastic deviations from a monotonic evolutionary trajectory due to the vagaries of random drift and recombination. Theory does identify scenarios in which competing sympatric populations coevolving under frequency‐dependent selection will exhibit nonmonotonic evolutionary trajectories in resource use traits (Fox & Vasseur, 2008). But in those scenarios trait evolution is relatively slow and nonmonotonic evolution arises because species abundances approach an equilibrium conditional on the current trait values. The resulting selection pressures eventually shift the trait values, changing the ecological equilibrium, leading to rapid change in species abundances as they approach the new equilibrium. We did not observe nonmonotonic changes in species’ absolute and relative abundances, only in their trait values, so a scenario like the one considered by Fox & Vasseur (2008) likely does not describe our experiment. Another possible explanation for nonmonotonic evolution is breakdown of linkage disequilibrium due to sexual reproduction, freeing a trait to return to its optimum value after having been pulled away from its optimum by selection on a linked trait (Falconer, 1960). However, breakdown of linkage disequilibrium is unlikely to explain nonmonotonic evolution in our study, due to weak phenotypic covariance between the assayed traits (all *R* ^2^ between assayed traits < .2). The reasons for the nonmonotonic evolution are unclear; exploring them would be an interesting direction for future work.

Our results on trait evolution broadly align with previously reported results on the coevolution of mutual invasibility in this experiment (Hausch et al., 2017). Mutual invasibility occurs when each competing species exhibits a positive per‐capita growth rate when rare. Species exhibiting mutual invasibility are expected to stably coexist in the long run (Chesson, 2000). Evolutionary character displacement should strengthen pre‐existing mutual invasibility or create mutual invasibility that did not previously exist, by weakening interspecific competition relative to intraspecific competition. However, in this experiment *C. chinensis* evolved improved ability to invade resident populations of *C. maculatus*, whether it was growing in allopatry or in sympatry with *C. maculatus*. In contrast, *C. maculatus* did not evolve improved ability to invade resident populations of *C. chinensis*. These results on the evolution of mutual invasibility are consistent with a scenario in which *C. chinensis* adapted primarily to aspects of the culture conditions that were shared among all treatments and did so by improving its ability to use lentils without much if any cost to its ability to use adzuki beans.

Our study adds to this list of experimental evolution studies that failed to detect strong ecological character displacement. The current empirical character displacement literature is dominated by studies testing if the mechanism of character displacement is responsible for observed divergence (Dayan & Simberloff, 2005; Schluter, 2000; Stuart & Losos, 2013). There are fewer tests of whether character displacement evolves under conditions that would seem favorable to its evolution, and most of those tests published since Taper (1990) are negative. Tyerman et al. (2008) found strong convergence in response to competitive release but only weak divergence with the reintroduction of competition. terHorst (2011) found divergence in one protozoan trait (growth rate) and convergence in another (cell size) but these changes produced no detectable local adaptation to the presence or absence of the competitor. Le Gac et al. (2012) initially observed divergence in resource use of two competing *E. coli* lineages, but further coevolution reduced the divergence rather than increasing it and one lineage eventually excluded the other. Bailey and Kassen (2012) did not detect higher niche diversification (individual resource use specialization) in heterogeneous environments, as would be expected due to character displacement. Theoretical models of character displacement, too, often do not produce strong character displacement, and predicted character displacement often is not robust to modest changes in model assumptions (Abrams, 1986; Jasmin & Kassen, 2007; Slatkin, 1980; Fox & Vasseur, 2008). Well‐established examples of character displacement in nature may be rare not only because investigators of putative cases of displacement rarely rule out all alternative explanations (Stuart & Losos, 2013), but because displacement occurs only under quite specific and uncommon conditions.

## CONFLICT OF INTEREST

None declared.

## AUTHOR CONTRIBUTION

SJH conceived and designed the experiment with advice from SMV and JWF. SJH collected and analyzed the data. SJH wrote the paper with assistance from JWF and SMV.

## Data Availability

Data available from the Dryad Digital Repository: https://doi.org/10.5061/dryad.3r2280gcn.
